# Distant and Distressed: Interpersonal Problems in Anxiety and Depression

**DOI:** 10.1002/jclp.70212

**Published:** 2026-09-12

**Authors:** Jacqueline Peters, Peter J. de Jong, Marije aan het Rot

**Affiliations:** ^1^ Department of Psychology University of Groningen Groningen the Netherlands

**Keywords:** anxiety disorders, chronicity, comorbidity, depression, interpersonal problems, interpersonal theory

## Abstract

**Objectives:**

Interpersonal functioning has a complex, bidirectional relationship with mental disorders such as anxiety and depression. The Inventory of Interpersonal Problems (IIP) assesses aspects of interpersonal functioning, including general interpersonal distress and interpersonal styles. In this study, we aimed to better understand how these aspects relate to anxiety and depression by (1) comparing interpersonal distress and style in individuals with current, past, and no lifetime anxiety and/or depression, and (2) examining how interpersonal styles relate to the comorbidity and chronicity of anxiety and depression.

**Methods:**

Participants (*N* = 1800) were drawn from the 9‐year follow‐up of the *Netherlands Study of Depression and Anxiety*. Diagnostic data from baseline until the 9‐year follow‐up were used to determine diagnoses and chronicity. General interpersonal distress and style were derived from the IIP using the Structural Summary Method. Participants were grouped into four interpersonal style subtypes corresponding to the quadrants of the interpersonal circumplex.

**Results:**

Currently diagnosed participants reported higher general interpersonal distress and a more submissive style than those with past and no lifetime anxiety/depression. As predicted, a cold‐submissive style was associated with greater comorbidity and chronicity, also after adjusting for demographics.

**Conclusions:**

This study extends prior research on how patients' interpersonal style relates to psychopathology by using longitudinal, transdiagnostic data of individuals across different symptomatic stages. Our findings indicate that a cold‐submissive style is relatively common in more comorbid and chronic anxiety and depressive disorders. This highlights the clinical value of integrating interpersonal assessment into treatment planning.

## 1 Introduction

The ability to interact meaningfully with others across different social contexts is a crucial aspect of psychological wellbeing. According to Contemporary Integrative Interpersonal Theory, interpersonal problems arise when a person's way of interacting with others, i.e., their interpersonal style, does not match their social environment for a sustained period of time (Wright et al. 2023). These interpersonal problems are then believed to contribute to anxiety and depression (Alden and Regambal 2012; Hames et al. 2013). Indeed, anxiety and depressive disorders have, at a group level, been associated with heightened interpersonal distress and an overly submissive interpersonal style compared to normative samples (Barrett and Barber 2007; Cain et al. 2010; Grosse Holtforth et al. 2014). There is interpersonal heterogeneity within specific disorders however (e.g., Cain et al. 2010, 2012), which is important because preliminary research suggests that the chronicity of anxiety and depressive disorders may differ between subtypes of interpersonal styles (Cain et al. 2012), hereafter *interpersonal subtypes*. In this study, we took a transdiagnostic approach to better understand how interpersonal problems relate to anxiety and depression by contrasting general interpersonal distress and interpersonal style in those with current, past, and no lifetime diagnoses of anxiety and/or depressive disorders, and by examining which interpersonal styles are associated with greater comorbidity as well as chronicity of anxiety and depression.

### 1.1 Interpersonal Circumplex Model

Interpersonal style, along with interpersonal distress, is a central aspect of interpersonal functioning and can be conceptualized with the *interpersonal circumplex model* (Leary 1957; Wiggins 1996), which is rooted in interpersonal theory (Sullivan 1953; Wright et al. 2023). In this model, interpersonal styles and associated behavioral tendencies are reflected by a combination of the orthogonal dimensions of Agency and Communion (Hopwood et al. 2013, 2021; Wright et al. 2023). Agency represents a tendency to exert dominance or assertion as opposed to passivity or submission. Communion reflects a tendency to engage in friendly interactions with others or warmth, as opposed to interpersonal distance or coldness. The combination of Agency and Communion can be visualized in the circular space of the interpersonal circumplex (S1). Interpersonal styles within this space can be explored using a dimensional (Wendt et al. 2019) or a categorical approach, for example by splitting the circumplex space into octants (Alden et al. 1990) or quadrants (Gurtman 1996). Interpersonal quadrants reflect warm‐dominant (Q1, 0–90°), cold‐dominant (Q2, 90–180°), cold‐submissive (Q3, 180–270°), and warm‐submissive (Q4, 270–360°) interpersonal styles (S1).

### 1.2 Interpersonal Pathoplasticity

On a group level, individuals with anxiety and depression tend to be more submissive than normative samples (e.g., Barrett and Barber 2007; Cain et al. 2010; Grosse Holtforth et al. 2014; Salzer et al. 2010). However, within specific diagnoses there is between‐person heterogeneity (Cain et al. 2010, 2012; Girard et al. 2017). Two people with depression may thus engage in different problematic interpersonal styles and associated behaviors, such as avoiding others, reflecting a cold interpersonal style, versus relying overly on support, which reflects a warm interpersonal style. Conversely, two individuals with similar interpersonal styles may exhibit different symptoms, leading to distinct diagnoses. In short, not all anxious or depressed individuals have similar problematic interpersonal styles and not all individuals with comparable interpersonal styles will exhibit similar symptoms. This phenomenon, known as *interpersonal pathoplasticity*, implies that a person's interpersonal pattern of interacting with others can influence the expression and trajectory of psychopathology and vice versa (Cain et al. 2010, 2012; Girard et al. 2017). Importantly, while interpersonal pathoplasticity assumes a bidirectional relationship between the presentation of interpersonal problems and psychopathology, neither fully causes the other (Cain et al. 2012; Klein et al. 1993; Widiger 2011).

The interpersonal pathoplasticity within depression and anxiety disorders has previously been investigated using cluster analysis to group individuals with similar interpersonal styles. The number of interpersonal subtypes that resulted from these analyses varied across studies and disorders. In individuals with depression, between two and eight subtypes were identified (Cain et al. 2012; Grosse Holtforth et al. 2012, 2014; Locke et al. 2018; McMillen et al. 2021; Simon et al. 2015). Two or three interpersonal subtypes were found in socially anxious individuals (Cain et al. 2010; Cooper and Anderson 2019; Frandsen et al. 2020), four interpersonal subtypes in generalized anxiety disorder (GAD; Przeworski et al. 2011; Salzer et al. 2008, 2011), mixed anxiety disorders (Pitman and Hilsenroth 2016), as well as posttraumatic stress disorder (PTSD; Thomas et al. 2014), and two interpersonal subtypes in panic disorder (Zilcha‐Mano et al. 2015).

Although these findings may suggest that depression is more interpersonally heterogeneous than anxiety, the variation in the number of identified interpersonal subtypes may also reflect differences in statistical methods. For example, Grosse Holtforth et al. (2014) explored a large range of cluster solutions (i.e., 1–11), whereas Cain et al. (2010) and Gómez Penedo et al. (2019) focused on a narrower range of possible interpersonal subtypes (i.e., 2–4). Additionally, cluster analysis is often data set specific and can be sensitive to sample size and characteristics, limiting generalizability. Therefore, a quadrant‐based split has been proposed as an alternative categorical approach (Gurtman 1996; König et al. 2016). This approach offers several advantages: It is not dependent on sample characteristics and avoids the data set‐specific variability inherent to cluster solutions, and it therefore allows direct comparisons between interpersonal subtypes across data sets (König et al. 2016). Furthermore, quadrant membership is determined by each individual's actual position on the circumplex rather than by the composition of the sample, which means that categorization is stable regardless of who else is in the data set. The quadrant‐based approach can therefore also be applied to individual cases (König et al. 2016). Moreover, it is clinically intuitive and practical: The quadrants' boundaries are derived from the circumplex structure itself, split along the axes of Agency and Communion (Gurtman 1996; König et al. 2016), which facilitates visualization and communication with patients.

Irrespective of the precise number of identified interpersonal subtypes, a relatively consistent finding was that these subtypes typically did not differ in clinical presentation (e.g., symptom severity and comorbidity) and demographics (Cain et al. 2010; Kachin et al. 2001; Przeworski et al. 2011; Salzer et al. 2008). Exceptions include gender differences (e.g., Grosse Holtforth et al. 2014; Locke et al. 2018; Simon et al. 2015) and symptom differences (Frandsen et al. 2020; Grosse Holtforth et al. 2014; König et al. 2016) between interpersonal subtypes reported in some studies. Additionally, not all studies tested for subtype differences in comorbidity (Gómez Penedo et al. 2019; Grosse Holtforth et al. 2014; König et al. 2016; McMillen et al. 2021; Pitman and Hilsenroth 2016). Findings were more mixed regarding general interpersonal distress, where some studies did not find differences between interpersonal subtypes in this variable (Cain et al. 2010; Przeworski et al. 2011; Salzer et al. 2008, 2011; Zilcha‐Mano et al. 2015), whereas others reported differences in general interpersonal distress (Frandsen et al. 2020; König et al. 2016) or did not formally test for such differences (Cain et al. 2012; Gómez Penedo et al. 2019; Grosse Holtforth et al. 2014; Locke et al. 2018; McMillen et al. 2021; Pitman and Hilsenroth 2016; Simon et al. 2015). Taken together, differences in interpersonal subtypes at least do not seem to be fully explained by demographic or clinical factors, which emphasizes the importance of examining how different interpersonal styles relate to anxiety and depression over time.

Preliminary evidence from the few longitudinal studies to date suggests that interpersonal styles may predict symptom trajectories. Regarding the chronicity of symptoms, Cain et al. (2012) found that individuals with major depressive disorder with a submissive subtype (located in the cold‐submissive quadrant, Q3) were more chronically depressed over the course of the following 10 years than all four interpersonal subtypes in the remaining three quadrants of the interpersonal circumplex. Only the cold subtype (also located in Q3) did not differ in chronicity from the submissive subtype. Similarly, a cold‐submissive interpersonal style was associated with more chronic PTSD compared to a warm‐dominant or cold‐dominant interpersonal style (Thomas et al. 2014). In terms of treatment response, a cold‐submissive or cold interpersonal style seems to be associated with relatively bad treatment outcomes across various anxiety and mood disorders (Cain et al. 2010; Gómez Penedo et al. 2019; Salzer et al. 2011).

Cumulatively, these previous studies indicate that specific disorders are characterized by interpersonal heterogeneity, and that some interpersonal styles may predict less favorable disorder trajectories. However, most studies were cross‐sectional, focused on a single, current diagnosis, and sample sizes tended to be relatively small (range 31–508). If specific interpersonal styles could predict chronicity and relapse beyond diagnostic categories, then interpersonal assessment has clear clinical relevance: It could inform prognosis and help identify individuals who may benefit from additional support, regardless of their DSM diagnosis. A naturalistic study with a large sample size, including longitudinal data on both current and lifetime anxiety and depressive disorders and a comparison group of individuals never diagnosed with anxiety or depression would be a next step in advancing our understanding of the role of interpersonal functioning in the course of anxiety and depression.

### 1.3 Netherlands Study of Depression and Anxiety

The Netherlands Study of Depression and Anxiety (NESDA; nesda.nl) is a longitudinal, naturalistic cohort study that allows for such a transdiagnostic approach as it examines several anxiety disorders (i.e., GAD, social phobia, panic disorder, agoraphobia) and two depressive disorders (i.e., major depressive disorder and dysthymia). The baseline assessment (2004–2007) included 2981 participants who had a current anxiety and/or depressive disorder or who were at risk for depression and anxiety (e.g., lifetime diagnosis), as well as a comparison group of individuals who had no history of anxiety or depression (see Penninx et al. 2008 for details). Due to its longitudinal design and large sample size, NESDA is well‐suited to investigate the role of problematic interpersonal styles in the presentation and course of anxiety and depression. Its transdiagnostic scope also allows for monitoring the chronicity of anxiety and depression, regardless of specific diagnoses. This seems especially relevant because new disorders can emerge over time and only focusing on the chronicity of the index disorder may not reflect clinically meaningful recovery (Remmerswaal et al. 2024; Scholten et al. 2016).

### 1.4 Research Aims and Hypotheses

In the current study, we took a transdiagnostic approach to better understand the role of interpersonal problems in the course of anxiety and depression, using the 9‐year follow‐up of the NESDA (i.e., wave 6, when interpersonal functioning was first assessed) as well as diagnostic data since the baseline assessment. To this end, we first characterized the interpersonal functioning, including general interpersonal distress and style, of individuals from wave 6 with a current diagnosis (*n* = 371), a past diagnosis (*n* = 1063), or no lifetime diagnosis (*n* = 366) of an anxiety and/or depressive disorder^1^. In line with previous research, we expected that individuals with a current diagnosis of anxiety and/or depression experienced greater general interpersonal distress (Hypothesis 1) and engaged in a more submissive interpersonal style (Hypothesis 2) on average compared to those without a history of anxiety or depression (i.e., the comparison group).

To better understand interpersonal pathoplasticity from a transdiagnostic perspective, we also examined how different interpersonal styles relate to the presentation and course of psychopathology. It is a strength of the present study that NESDA includes individuals with various current and past anxiety and depressive disorders, as well as a comparison group that was never diagnosed with any of the examined anxiety or depressive disorders. This can offer insights into interpersonal pathoplasticity beyond diagnostic categories. Similar to Gurtman (1996), we chose a quadrant‐based approach to categorize individuals into interpersonal subtypes instead of cluster analysis to improve interpretability and comparability across data sets and to facilitate straightforward illustration of different styles in clinical practice. Previous clinical research tentatively suggests more chronic symptom trajectories (Cain et al. 2012; Thomas et al. 2014) and poorer treatment response (Cain et al. 2010; Gómez Penedo et al. 2019; Salzer et al. 2011) in individuals with a cold‐submissive interpersonal style (Q3). However, to date only one naturalistic longitudinal study has investigated the link between interpersonal style and disorder chronicity in one of the disorders examined in the NESDA, focusing on major depression (Cain et al. 2012). Moreover, little is known about how interpersonal style is associated with clinical outcomes in people who are at risk for relapse but not currently meeting diagnostic criteria. Additionally, differences in comorbidity between interpersonal styles were often not assessed. Given that chronicity and comorbidity are associated (e.g., Klein Hofmeijer‐Sevink et al. 2012), we predicted that the proportion of comorbid anxiety and depression would be highest in the cold‐submissive quadrant (Q3; Hypothesis 3). Lastly, we expected that cold‐submissive individuals experienced more *chronic* anxiety and depression than other interpersonal subtypes (Hypothesis 4).

Taken together, the present study expands the previous literature in several ways: First, we examined interpersonal distress and style in individuals with current, past, and no lifetime diagnoses across multiple anxiety and depressive disorders. Especially individuals with a past diagnosis, who may be vulnerable to relapse or experience residual symptoms, are an additional clinically meaningful group in this context. Exploring their interpersonal functioning may offer insights into whether interpersonal problems persist beyond acute episodes. Second, we examined the association between interpersonal style and the chronicity of anxiety and depression, which remains understudied. Third, by drawing on 9 years of longitudinal diagnostic data from NESDA, the present study complements previous smaller‐scale studies with a large, naturalistic data set that offers ecological validity. Fourth, the transdiagnostic approach of the present study, covering four anxiety and two depressive disorders, reflects a shift towards examining shared interpersonal correlates beyond diagnostic categories. Lastly, the present study extends a recently published study, in which we described the average interpersonal distress and style for each of the anxiety and depressive disorders in NESDA (Peters and aan het Rot 2026), by examining interpersonal heterogeneity and by including individuals with past and no lifetime diagnoses.

## 2 Methods

### 2.1 Participants

The present study included participants from the 9‐year follow‐up of NESDA (wave 6; 2013–2016), when interpersonal problems were first assessed, as well as diagnostic data from the baseline assessment, 2‐year follow‐up, 4‐year follow‐up, and 6‐year follow‐up to determine the chronicity of anxiety and depression. NESDA participants were recruited from the community, general practice, and mental health care. Inclusion criteria required Dutch fluency and no primary diagnosis of an obsessive‐compulsive disorder, psychotic disorder, bipolar disorder, or severe addiction. The NESDA was approved by the Medical Ethical Committee of the VUmc (reference number 2003/183), and written informed consent was obtained from all participants. For details on the recruitment process, study design, and objectives, see Penninx et al. (2008). At the 9‐year follow‐up, 2069 participants remained, indicating a good retention rate of 69%. Of these, 119 were excluded from the present study because they did not return self‐report questionnaires, one participant was excluded for missing 11 responses of the 32 items assessing interpersonal problems. Of the remaining 1949 participants, we excluded 149 individuals for having missed at least one follow‐up during which diagnostic data were obtained. This resulted in a final sample of 1800 participants^2^, aged 26–75 years (*M*_*age*_ = 51.23, SD = 13.19; 1189 female, 611 male)^3^. Of these, 371 individuals had a current diagnosis of at least one of the examined anxiety or depressive disorders, 1063 individuals had a past diagnosis (i.e., lifetime history, but no current diagnosis), and 366 participants were never diagnosed with any of the examined anxiety or depressive disorders and formed our comparison group. Diagnostic status was thus categorized as (1) current diagnosis, (2) past diagnosis, and (3) no lifetime diagnosis (i.e., comparison group). Of the 371 participants with a current diagnosis, 98 participants had at least one depressive disorder, 177 had at least one anxiety disorder, and 96 had comorbid anxiety and depression. See S2 for supporting information on specific DSM‐IV diagnoses.

### 2.2 Materials and Measures

#### 2.2.1 Presence of an Anxiety or Depressive Disorder

The *Composite International Diagnostic Interview* (CIDI; Wittchen 1994) was used to determine a current diagnosis according to the Diagnostic and Statistical Manual of Mental Disorders (DSM‐IV; American Psychiatric Association 2000). The CIDI was found to be reliable and valid for classifying anxiety and depressive disorders (Wittchen 1994). A computerized Dutch version was used during the interview, in which the algorithm provides a DSM‐IV diagnosis, and estimates the period during which a mental health condition was present (ter Smitten et al. 1998). In addition to the 9‐year follow‐up (wave 6), the CIDI was also administered at baseline (wave 1), 2‐year follow‐up (wave 3), 4‐year follow‐up (wave 4), and the 6‐year follow‐up (wave 5).

In the present study, a current diagnosis of an anxiety disorder (social phobia, generalized anxiety disorder, agoraphobia, and panic with or without agoraphobia) or a depressive disorder (major depression, dysthymia) was based on meeting the diagnostic criteria in the past month according to the CIDI. Similarly, comorbid anxiety and depression was based on comorbidity of at least one anxiety and depressive disorder in the past month. To determine the chronicity of anxiety and depression (hereafter *chronicity*), we assessed whether a depressive or anxiety disorder was present since the previous assessment point (e.g., between the 6‐year and 9‐year follow‐up; lifetime history at baseline) and summed assessments with disorder presence. As such, chronicity ranged from 0 to 5 and was ordinal in nature due to the uneven spacing of follow‐ups.

#### 2.2.2 Interpersonal Functioning

*Inventory of Interpersonal Problems* (Horowitz et al. 2000 *;* Vanheule et al. 2006). The IIP is a self‐report questionnaire that describes interpersonal style along the dimensions of Communion and Agency on a 5‐point Likert scale (0‐4) and can be divided into eight subscales that reflect the octants of the interpersonal circumplex (Alden et al. 1990), i.e., it is a circular measure. The IIP also assesses to what extent different interpersonal styles and associated behaviors are currently distressing (Horowitz 2005, p. 122). In NESDA, a validated Dutch translation of the IIP with 32 items (Horowitz et al. 2000) has been used, which was confirmed to follow a circumplex structure in both clinical and non‐clinical samples (aan het Rot and Peters 2026; Vanheule et al. 2006). The reliability of the IIP octant subscales in the present study was adequate to good, with Cronbach's α ranging from 0.72 to 0.88, with the exception of the domineering/control subscale (PA) that just fell short of adequate internal consistency with an α of 0.68.

#### 2.2.3 Severity of Depression and Anxiety

*Quick Inventory of Depressive Symptomatology* (QIDS; Rush et al. 2003, *ids‐qids.org*). The 16‐item QIDS (range 0–27) was used to assess the current severity of depressive symptoms on a 4‐point Likert scale, ranging from 0 (not at all) to 3 (severely). Individual scores of the 16 items included in the QIDS were converted into nine domain scores, which are then summed to a total score of 0–27. Participants completed the full Inventory of Depressive Symptomatology (IDS; Rush et al. 1996), from which the QIDS was derived. We used the QIDS score instead of the IDS because it is more specific to depressive symptoms and does not assess symptoms related to anxiety.

*Beck Anxiety Inventory* (BAI; Beck et al. 1988). The BAI assesses the severity of anxiety by asking participants to indicate to what extent they were bothered by 21 symptoms, such as nervousness, in the past week. The 21 symptoms are assessed on a 4‐point Likert scale, ranging from 0 (not at all) to 3 (severely). The BAI is highly internally consistent and has acceptable test‐retest reliability (Beck et al. 1988; Fydrich et al. 1992)^4^.

#### 2.2.4 Other Self‐Reported Measures

We report demographic variables on age, sex, and marital status.

### 2.3 Procedure

Participants completed self‐report questionnaires at home. The CIDI was administered by trained staff during participants' in‐person session, and demographics were collected as part of an in‐person interview as well. Travel expenses were reimbursed and participants received a €15 gift certificate. Published articles using NESDA data can be found on the NESDA website (nesda.nl).

### 2.4 Data Analysis

#### 2.4.1 Structural Summary Method (SSM)

We used the *structural summary method (SSM)* to reduce an individual or group pattern of IIP responses to three clinically relevant parameters on a sinusoidal curve: *elevation*, *displacement*, and *amplitude* (Gurtman 1992; Wright et al. 2009; Zimmermann and Wright 2017). Elevation is the mean level of this curve and represents general interpersonal distress. Angular displacement (0–360°) indicates the predominant interpersonal style or problem that an individual or group is leaning towards (e.g., high Communion and low Agency indicates a warm‐submissive interpersonal style (Q4)), and amplitude indicates the specificity of this interpersonal profile. In the present study, we used the *circumplex* package (Girard et al. 2024) in R to calculate SSM parameters and the dimensional scores of Agency and Communion. A group's elevation can be interpreted irrespective of goodness of fit of the modeled curve to the observed data (R^2^), but amplitude and displacement are only fully interpretable if the goodness‐of‐fit (R^2^) is adequate (> 0.70; Wright et al. 2009; Zimmermann and Wright 2017). Inadequate fit, i.e., low R^2^, indicates low prototypicality, which means that the interpersonal profile of the group is either ambiguous or too heterogeneous and thus does not fit the sinusoidal curve well (Wright et al. 2009). See Zimmermann and Wright (2017) for a discussion of the advantages of SSM for circumplex data.

#### 2.4.2 Planned Analyses

To test whether individuals with a current diagnosis experienced more general interpersonal distress than the comparison group, we tested whether these groups meaningfully differed in elevation (Hypothesis 1), using Bayesian regressions. Bayes Factors (BF_10_) quantify the evidence for the model including the main effect of group compared to the null model, and we opted for regression models as they allow the inclusion of covariates. These regressions were conducted with the *brms* package (Bürkner 2018) in R, unless specified otherwise. To test whether individuals with a current diagnosis engaged in a more submissive interpersonal style than the comparison group, we compared whether the displacement differed between these groups (H2), using a Bayesian circular regression with the *bpnreg* package (Cremers 2024) in R, which is suitable for circular outcome variables like angular displacement (Cremers et al. 2021).

After examining interpersonal functioning by diagnostic status, we investigated the association between a person's predominant interpersonal style and psychopathology. To this end, we categorized individuals into four interpersonal subtypes based on the quadrants of the interpersonal circumplex, similar to König et al. (2016). The four interpersonal subtypes reflect a warm‐dominant (Q1, 0–90°), cold‐dominant (Q2, 90–180°), cold‐submissive (Q3, 180–270°), and warm‐submissive (Q4, 270–360°) interpersonal style. We then tested whether individuals with a cold‐submissive interpersonal style (Q3) were more likely to experience comorbid anxiety and depression compared to participants in the other three interpersonal quadrants, using a Bayesian multinomial logistic regression (Hypothesis 3). Next, we examined whether cold‐submissive individuals (Q3) experienced more chronic anxiety and depression than other interpersonal subtypes (Hypothesis 4), by conducting a Bayesian ordinal regression with chronicity as the outcome variable and quadrant‐based interpersonal subtypes (Q1–Q4) as the categorical predictor.

## 3 Results

### 3.1 Outliers and Missing Data

We did not exclude outliers as all values were considered plausible, but report extreme outliers exceeding three times the interquartile range for the IIP derived dimensions of Agency and Communion, general interpersonal distress (i.e., elevation), the QIDS, the BAI, and chronicity. There were no extreme outliers for Agency, elevation, the QIDS, or chronicity; neither for the full sample, nor when split by diagnostic status (S3). There were seven extreme outliers exceeding three times the interquartile range for the BAI and one for Communion.

There were no missing demographic data for age, sex, or marital status. For the QIDS and BAI, participants were excluded from the relevant analyses if more than six out of 28 items were missing for the IDS and if more than eight out of 21 items were missing for the BAI, in line with previous NESDA studies (e.g., Duivis et al. 2013; van Sprang et al. 2023), resulting in 0 and 5 missing QIDS and BAI scores, respectively.

### 3.2 Demographics and Descriptives

Descriptives of age, BAI, QIDS, Agency, and Communion scores are in Table 1, split by (A) diagnostic status as well as (B) quadrant‐based interpersonal style, and in S4 for the subset of participants with a current diagnosis. Whenever we detected meaningful group differences in demographic variables, we included them as covariates in our confirmatory analyses to assess whether the pattern of the results remained stable. For further exploration, we provide Spearman's correlations of general interpersonal distress (= elevation) with the symptom severity of depression (QIDS) and anxiety (BAI), as well as chronicity, Agency, and Communion in the supplement (S5). Additionally, correlation‐based SSM profiles for the BAI and QIDS scores are reported in S6. Both symptom severity measures were reflected by a submissive interpersonal profile, but the QIDS was associated with greater elevation than the BAI.

**Table 1:** Means (M) and standard deviations (SD) of age, symptom severity, chronicity, Agency and Communion, split by (A) diagnostic status and (B) quadrant‐based interpersonal style.

A. Diagnostic Status		Current diagnosis	Past diagnosis	Never diagnosed	BF_10_
Age	M (SD)	51.25 (11.81)	51.4 (13.15)	50.74 (14.61)	0.033
	*n*	*n* = 371	*n* = 1063	*n* = 366	
QIDS	M (SD)	9.97 (5.02)	5.13 (3.73)	2.57 (2.2)	5.13 × 10^131^
	*n*	*n* = 371	*n* = 1063	*n* = 366	
BAI	M (SD)	15.08 (9.97)	6.45 (6.64)	2.36 (3.4)	2.08 × 10^117^
	*n*	*n* = 369	*n* = 1060	*n* = 366	
Chronicity	M (SD)	4.1 (1.1)	2.33 (1.29)	n/a	2.959 × 10^6^*
	*n*	*n* = 371	*n* = 1063		
Agency	M (SD)	−0.54 (0.51)	−0.32 (0.41)	−0.16 (0.32)	8.29 × 10^27^
	*n*	*n* = 371	*n* = 1063	*n* = 366	
Communion	M (SD)	0.26 (0.55)	0.27 (0.48)	0.21 (0.36)	2.73 × 10^−^^4^
	*n*	*n* = 371	*n* = 1063	*n* = 366	

B. Interpersonal Style		Q1: Warm‐dominant	Q2: Cold‐dominant	Q3: Cold‐submissive	Q4: Warm‐submissive	BF_10_
Age	M (SD)	51.46 (13.17)	52.64 (12.63)	53.32 (12.79)	50.39 (13.3)	7.69
	*n*	*n* = 299	*n* = 78	*n* = 348	*n* = 1075	
QIDS	M (SD)	4.35 (3.99)	5.28 (3.8)	7.03 (4.89)	5.52 (4.44)	2.19 × 10^9^
	*n*	*n* = 299	*n* = 78	*n* = 348	*n* = 1075	
BAI	M (SD)	5.36 (7.56)	6.21 (6.68)	9.03 (8.77)	7.51 (8.1)	40668.48
	*n*	*n* = 298	*n* = 78	*n* = 346	*n* = 1073	
Chronicity	M (SD)	1.6 (1.6)	2.04 (1.64)	2.78 (1.78)	2.22 (1.68)	9.87 × 10^15^*
	*n*	*n* = 299	*n* = 78	*n* = 348	*n* = 1075	
Agency	M (SD)	0.17 (0.17)	0.23 (0.18)	−0.51 (0.38)	−0.46 (0.37)	3.41 × 10^179^
	*n*	*n* = 299	*n* = 78	*n* = 348	*n* = 1075	
Communion	M (SD)	0.41 (0.33)	−0.28 (0.27)	−0.36 (0.32)	0.45 (0.35)	1.08 × 10^261^
	*n*	*n* = 299	*n* = 78	*n* = 348	*n* = 1075	

*Note:* Values are means with standard deviations in parentheses. *n* = number of participants per group. QIDS: Quick Inventory of Depressive Symptomatology (range: 0–27). BAI: Beck Anxiety Inventory (range: 0–63). Chronicity: Number of follow‐ups where anxiety and/or depressive disorder diagnosis was present between waves (range 0–5). Dimensional scores of Agency and Communion. Diagnostic status indicates current, past, or no lifetime diagnosis of any of the examined anxiety and depressive disorders. BF_10_: Bayes Factor. BF_10_ > 1 indicates that the data are more likely under the model including a main effect compared to under the null model, using Bayesian regressions. For example, for age the data are 7.69 times more likely under the model that includes a main effect when it comes to group differences in quadrant‐based interpersonal style. *BF_10_ for Chronicity is based on a Mann‐Whitney *U* test (A) and a Bayesian ordinal regression model (B) due to the ordinal nature of this variable. Only those with a current and past diagnosis were considered for this comparison in (A) as chronicity for those never diagnosed is always zero.

Whereas symptom severity differed by diagnostic status in the full sample (BAI: BF_10_ = 2.08 × 10^117^; QIDS: BF_10_ = 5.13 × 10^131^), we did not find evidence for differences in symptom severity in the currently diagnosed subset (BAI: BF_10_ = 0.11; QIDS: BF_10_ = 0.08).

For categorical demographic variables, Bayesian logistic regression models indicated strong evidence for the inclusion of a main effect of diagnostic status for both sex (BF_10_ = 115.82) and marital status (BF_10_ = 47950.73). We also found strong evidence for a main effect of quadrant‐based interpersonal style for sex (BF_10_ = 3.70 × 10^23^) and marital status (BF_10_ = 3142.19). See S7 for frequency tables of sex and marital status, split by diagnostic status as well as interpersonal quadrant.

### 3.3 Results on Interpersonal Functioning by Diagnostic Status (Hypotheses 1 and 2)

The SSM parameters elevation, amplitude, and displacement, split by current diagnostic status are in Table 2A, and the respective radar chart is in Figure 1. See S8 for results on the structural validity of the IIP in our sample.

**Table 2:** Mean‐based structural summary statistics with 95% confidence intervals, split by (A) current diagnostic status and (B) quadrant‐based interpersonal style.

	N	Elevation	Amplitude	Displacement	Fit (R^2^)
(A) Diagnostic Status					
Current diagnosis	371	1.37 (1.31, 1.43)	0.60 (0.55, 0.66)	295.6 (290.4, 300.6)	0.895
Past diagnosis	1063	0.93 (0.89, 0.96)	0.42 (0.39, 0.45)	310.4 (306.6, 313.8)	0.887
Never diagnosed	366	0.54 (0.49, 0.58)	0.27 (0.23, 0.31)	322.2 (314.7, 329.1)	0.852
(B) Interpersonal Style					
Q1: Warm‐dominant	299	0.63 (0.58, 0.69)	0.44 (0.41, 0.48)	22.4 (19.7, 25.1)	0.941
Q2: Cold‐dominant	78	1.08 (0.94, 1.22)	0.37 (0.31, 0.42)	140.4 (132.6, 147.6)	0.648
Q3: Cold‐submissive	348	1.30 (1.24, 1.37)	0.62 (0.58, 0.66)	234.8 (231.9, 237.8)	0.830
Q4: Warm‐submissive	1075	0.90 (0.87, 0.93)	0.65 (0.62, 0.67)	314.6 (312.9, 316.2)	0.953

*Note:* N indicates sample size per group. Diagnostic status indicates current, past, or no lifetime diagnosis of any of the examined anxiety and depressive disorders. Structural Summary Statistics are based on the Inventory of Interpersonal Problems. R^2^: Goodness of fit of the modeled sinusoidal curve to the observed data.

**Figure 1:**
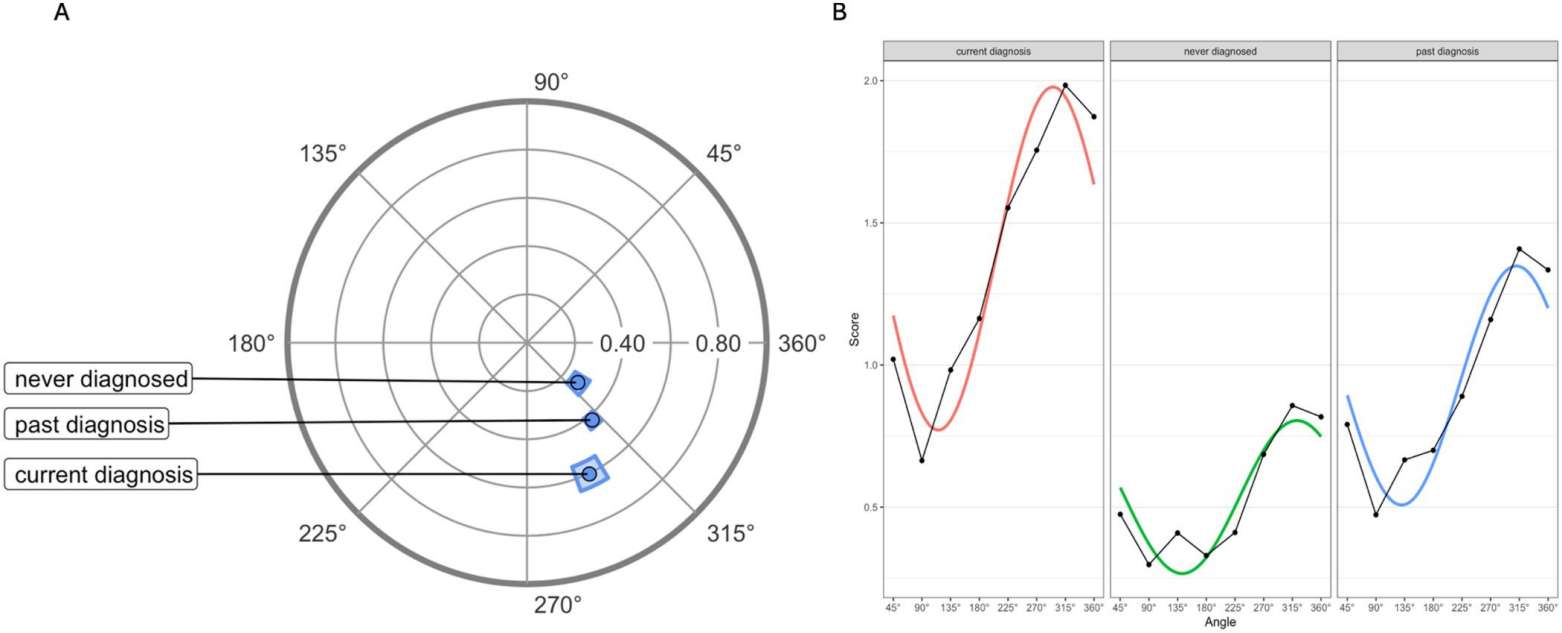
Radar chart (A) and curve plot (B) of diagnostic status projected into the Interpersonal Circumplex Model. Based on the Inventory of Interpersonal Problems. Current diagnosis: *n* = 371. Past diagnosis: *n* = 1063. Never diagnosed (no lifetime diagnosis of the examined disorders): *n* = 366. (A) Dots represent mean Amplitude and Displacement values and colored regions reflect the corresponding 95% confidence intervals. (B) Curve plots indicate the goodness of fit of the modeled to the observed data.

Bayesian regression model comparisons indicated group differences in elevation based on diagnostic status by favoring the model including a main effect of group compared to the null model (BF_10_ = 2.87 × 10^83^). As expected, individuals with a current diagnosis experienced greater elevation than the comparison group, with a difference in elevation of −0.84 (95% credible interval [CI] [−0.92, −0.76], confirming hypothesis 1), and than those with a past diagnosis with a difference of −0.45 (95% CI [−0.51, −0.38]). The effects of diagnostic status on elevation remained stable when adding sex or marital status as covariates. The fit to the modeled curve was good across diagnostic groups (R^2^ > 0.85).

Whereas all three diagnostic groups showed a warm‐submissive interpersonal style on average, a circular regression showed that—in line with hypothesis 2—individuals with a current diagnosis reported greater submissiveness than the comparison group (Δ = −0.59, 95% CI [ − 0.73, −0.44]). Displacement also differed between those with a current and past diagnosis (Δ = −0.30, 95% CI [ − 0.42, −0.19]), as well as between those with a past diagnosis and the comparison group (Δ = −0.87, 95% CI [ − 1.07, −0.65]). The effect of diagnostic status on angular displacement remained stable when controlling for sex and marital status.

### 3.4 Results on Quadrant‐Based Interpersonal Styles

Interpersonal subtypes by quadrant were based on each individual's angular displacement (S1). See Table 1B for demographics, symptom severity, chronicity, as well as Agency and Communion, split by interpersonal quadrant, and Table 2B for quadrant‐based SSM parameters. The fit to the modeled curve was good (R^2^ > 0.83), except for an inadequate fit for Q2 (cold‐dominant; R^2^ = 0.648). A radar chart and curve plot of quadrant‐based interpersonal functioning can be found in S9.

#### 3.4.1 Is a Cold‐Submissive Interpersonal Style Associated With Greater Comorbidity? (Hypothesis 3)

For the percentage of individuals in each quadrant who did not have a current diagnosis, or who were currently diagnosed with an anxiety disorder, depressive disorder, or comorbid anxiety and depression see Table 3. A Bayesian multinomial logistic regression indicated that individuals with a cold‐submissive interpersonal style (Q3) were more likely to have comorbid anxiety and depression than the remaining three quadrants in the full sample (Q3 and “no current diagnosis” as reference groups; Q1: Δ = –1.28, 95% CI [–2.07, –0.56]; Q2: Δ = –1.79, 95% CI [–3.66, –0.42]; Q4: Δ = –0.80, 95% CI [–1.26, –0.34]). Expressed as odds ratios (OR), Q3 showed greater odds of comorbid anxiety and depression relative to no current diagnosis compared to Q1 (OR = 3.54, 95% CI [1.76, 7.79]), Q2 (OR = 5.52, 95% CI [1.51, 39]), and Q4 (OR = 2.24, 95% CI [1.41, 3.52]). When including age, sex, or marital status as covariates in the model as sensitivity analyses, these results remained similar, supporting their stability. Additionally, Q3 was also more likely to only have an anxiety disorder compared to Q1 (Δ = −1.15, 95% CI [−1.73, −0.59]; OR = 3.14, 95% CI [1.81, 5.67]), Q2 (Δ = −3.26, 95% CI [−6.28, −1.35]; OR = 21.1, 95% CI [3.82, 513]), and Q4 (Δ = –0.56, 95% CI [−0.91, −0.20]; OR = 1.75, 95% CI [1.22, 2.49]). Q3 did not differ from the other quadrants in the likelihood of depression‐only.

**Table 3:** Current diagnosis by quadrant‐based interpersonal style.

Interpersonal Style	No diagnosis	Depression‐only	Anxiety‐only	Comorbid
Q1: Warm‐dominant	259 (86.6%)	12 (4%)	18 (6%)	10 (3.3%)
Q2: Cold‐dominant	70 (89.7%)	5 (6.4%)	1 (1.3%)	2 (2.6%)
Q3: Cold‐submissive	246 (70.7%)	16 (4.6%)	53 (15.2%)	33 (9.5%)
Q4: Warm‐submissive	854 (79.4%)	65 (6%)	105 (9.8%)	51 (4.7%)

*Note:* Values represent number of participants per cell: *n* (percentage within quadrant). No diagnosis: No current diagnosis of the examined anxiety and depressive disorders. Depression‐only: Current diagnosis of dysthymia, major depressive disorder, or both, but no anxiety disorder. Anxiety‐only: Current diagnosis of at least one anxiety disorder (i.e., social phobia, panic disorder with or without agoraphobia, agoraphobia without panic disorder, or generalized anxiety disorder), but no depressive disorder. Comorbid: Current comorbid anxiety and depression, i.e., at least one anxiety and depressive disorder. Diagnostic status was determined based on the Composite International Diagnostic Interview. Current diagnosis was defined as meeting the diagnostic criteria in the past month.

Of note, when only including the currently diagnosed subset (i.e., excluding the “no current diagnosis” category), then Q3 was more likely to experience comorbid anxiety and depression than depression alone compared to Q2 (Δ = 1.82, 95% CI [0.07, 3.91]; OR = 5.88, 95% CI [1.07, 50.1]) and Q4 (Δ = 0.99, 95% CI [0.29, 1.71]; OR = 2.67, 95% CI [1.33, 5.51]), but not Q1 (Δ = 0.93, 95% CI [‐0.10, 1.98]; OR = 2.53, 95% CI [0.90, 7.26]). There were no credible differences between quadrants regarding the likelihood of experiencing comorbid anxiety and depression compared to anxiety alone (Q3 as reference group; Q1: Δ = 0.13, 95% CI [–0.77, 1.06]; OR = 1.14, 95% CI [0.47, 2.89]; Q2: Δ = ‐1.48, 95% CI [–4.87, 1.22]; OR = 0.25, 95% CI [0.01, 3.37]; Q4: Δ = 0.25, 95% CI [–0.31, 0.79]; OR = 1.28, 95% CI [0.74, 2.21]). The Q2 estimates should be interpreted with caution given the wide credible intervals.

#### 3.4.2 Is a Cold‐Submissive Interpersonal Style Associated With Greater Chronicity? (Hypothesis 4)

A Bayesian ordinal regression indicated that, in the full sample, individuals with a cold‐submissive interpersonal style (Q3) showed greater chronicity of disorder compared to the remaining three quadrants, and there was very strong evidence for the inclusion of a main effect of quadrant (BF_10_ = 9.87 × 10^15^). Specifically, Q3 showed greater chronicity compared to Q1 (warm‐dominant; Δ = 1.27, 95% CI [0.99, 1.55], OR = 3.57, 95% CI [2.68, 4.73]), Q2 (cold‐dominant; Δ = 0.77, 95% CI [0.35, 1.2], OR = 2.16, 95% CI [1.42, 3.33]), and Q4 (warm‐submissive; Δ = 0.59, 95% CI [0.37, 0.81], OR = 1.80, 95% CI [1.45, 2.24]). These effects remained stable in our sensitivity analyses, in which we controlled for age, sex, and marital status, providing support for the robustness of these findings.

Due to its clinical relevance, we also explored whether a cold‐submissive interpersonal style is associated with chronicity in the subset of participants with a current diagnosis. Bayesian regressions^5^ indicated evidence for the inclusion of a main effect of quadrant (BF_10_ = 20.18). Again, Q3 showed greater chronicity compared to Q1 (Δ = 0.55, 95% CI [0.16, 0.95]) and Q4 (Δ = 0.28, 95% CI [0.04, 0.52]). The difference to Q2 was large but not statistically meaningful (Δ = 0.73, 95% CI [‐0.01, 1.5]), likely due to the low number of individuals in Q2 (*n* = 8). Findings from analyses involving Q2 should therefore be interpreted with caution.

## 4 Discussion

In the present study, we examined the role of interpersonal problems in anxiety and depression using data from a large, longitudinal cohort. As important extensions of the existing literature, we examined interpersonal problems across different symptomatic stages by including a sample with current, past, and no lifetime diagnoses of anxiety and/or depression, rather than focusing on a single disorder in symptomatic patients (e.g., Cain et al. 2010; Cain et al. 2012; Locke et al. 2018; Salzer et al. 2008; Zilcha‐Mano et al. 2015). We also investigated how interpersonal style relates to the comorbidity and chronicity of anxiety and depression, using longitudinal data collected over the previous 9 years. We found that individuals with a current diagnosis reported more general interpersonal distress than the comparison group without a lifetime diagnosis of anxiety or depression (Hypothesis 1) and than those with a past diagnosis. While the average interpersonal style in our sample was warm‐submissive, individuals with a current diagnosis engaged in a more submissive interpersonal style than the comparison group (Hypothesis 2) and than those with a past diagnosis. These findings align with prior studies that reported heightened interpersonal distress and greater submissiveness in individuals with anxiety and depression compared to normative samples (Barrett and Barber 2007; Cain et al. 2010; Grosse Holtforth et al. 2014).

### 4.1 Evidence for Interpersonal Pathoplasticity

Although the average interpersonal style in our sample was warm‐submissive, there was interpersonal heterogeneity that spanned the entirety of the interpersonal circumplex (S1). This allowed us to explore how different interpersonal styles relate to anxiety and depression using a quadrant‐based approach. Compared with the other three quadrants, a cold‐submissive interpersonal style (Q3) was associated with greater comorbid anxiety and depression (Hypothesis 3), as well as greater chronicity of anxiety and depression (Hypothesis 4).

These findings are partially consistent with the concept of interpersonal pathoplasticity, a framework that can help us understand how mental disorders are expressed and maintained differently based on a (mis)match of an individual's interpersonal style with their social environment. In its strictest form, evidence for interpersonal pathoplasticity requires (1) interpersonal heterogeneity within the sample, (2) no interpersonal subtype differences in relevant demographic or clinical variables, such as pre‐treatment symptom severity, comorbidity, and/or general interpersonal distress, and (3) different disorder trajectories (e.g., chronicity) across subtypes (Cain et al. 2010, 2012).

In the subset with a current diagnosis, (1) interpersonal heterogeneity was present (S1). Second, (2) interpersonal subtypes differed in general interpersonal distress, but not symptom severity. Additionally, there was some evidence for interpersonal subtype differences in the comorbidity of anxiety and depression. Third, (3) a cold‐submissive interpersonal style was associated with greater chronicity of anxiety and depression. Together, these findings provide some evidence for interpersonal pathoplasticity among individuals with current diagnoses. They broadly align with previous longitudinal studies on depression (Cain et al. 2012) and social phobia (Cain et al. 2010). Note that Cain et al. (2010) did not find interpersonal subtype differences in general interpersonal distress however, and Cain et al. (2012) did not examine interpersonal subtype differences in this variable. The present study, alongside Cain et al. (2010, 2012), is among the few studies that could test all three criteria of interpersonal pathoplasticity.

When extending our analyses to our full sample, interpersonal heterogeneity was also present and a cold‐submissive interpersonal style was associated with greater chronicity as well. In contrast to the currently diagnosed subset, we found strong evidence for symptom severity differences between interpersonal subtypes in the full sample. These differences in symptom severity may partly stem from a larger number of individuals with a current diagnosis in the submissive quadrants. We did not evaluate the criteria for interpersonal pathoplasticity for the full sample as this group included a substantial proportion of non‐symptomatic and sub‐clinical individuals. The variation in symptom severity depending on interpersonal style in the full sample may reflect differences in vulnerability, resilience, or remission trajectories. During an episode, the contribution of interpersonal style to symptom expression may be overshadowed by the acuteness of the episode, but importantly, interpersonal style was still uniquely associated with chronicity.

Overall, these findings support the idea that psychopathology and interpersonal functioning are partially independent dimensions, and their bidirectional association seems non‐linear, with less shared variance between the two phenomena during an active episode of disorder, as would be expected in the framework of interpersonal pathoplasticity. If replicated in prospective studies, this tentatively suggests that interpersonal style can be useful beyond symptom severity to identify people who are at risk for developing a disorder and for poorer long‐term outcomes.

### 4.2 Interpersonal Style Versus Comorbidity as a Chronicity Indicator?

Interpersonal heterogeneity has previously been interpreted as evidence of pathoplasticity as discussed above. Alternatively, it has also been interpreted as evidence of the influence of comorbidity on interpersonal problems (e.g., Barrett and Barber 2007), that is, the idea that an individual's problematic interpersonal style depends on co‐occurring disorders (see Girard et al. 2017). Comorbidity is itself associated with chronicity (Klein Hofmeijer‐Sevink et al. 2012). The association between a cold‐submissive style and a chronic course may potentially represent the additive or interacting effect of comorbid anxiety and depression, rather than a specific interpersonal style constituting a unique risk factor. Cain et al. (2010) argued that differences in comorbidity between interpersonal subtypes challenge the support for interpersonal pathoplasticity (i.e., criterion 2) as chronicity might be better explained by comorbidity than by interpersonal style. Our findings cannot exclude this possibility, as the cold‐submissive subtype showed the highest rates of comorbid anxiety and depression alongside a more chronic course of anxiety and depression, at least in the full sample. In those with a current diagnosis, a cold‐submissive interpersonal style remained strongly associated with chronicity, while the results were less clear for comorbid anxiety and depression. From a clinical perspective, interpersonal style may present a more concrete and helpful target for intervention than comorbidity (Cain et al. 2023).

### 4.3 Clinical Implications

Interpersonal assessment is clinically intuitive and practical in order to understand how interpersonal styles may affect symptom trajectories and treatment, and has thus been proposed complementary, or even alternative, to DSM‐based classification systems (Hopwood et al. 2021, 2023). In line with this, our findings and previous research (Cain et al. 2010, 2012) suggest that interpersonal assessment could help clinicians identify diagnosed individuals who are at risk for chronic or recurrent mental health problems, which is especially relevant because during an episode these patients may not be distinguishable based on symptom severity alone. Interpersonal assessments can also help clinicians interact effectively in treatment based on the patient's interpersonal style, and can help patients understand where their (interpersonal) distress stems from and how it affects the expression of anxiety or depressive symptoms.

Regarding responsiveness to treatment, a cold or cold‐submissive interpersonal style may be associated with worse treatment outcomes across various anxiety and mood disorders (Cain et al. 2010; Gómez Penedo et al. 2019; Salzer et al. 2011)^6^. This suggests that two patients with the same diagnosis may benefit from different interventions that are tailored to their dysfunctional interpersonal patterns (Hopwood et al. 2013; McFarquhar et al. 2018, 2023). A cold‐submissive interpersonal style might interfere with recovery by limiting individuals' ability to seek support from their social environment, for example through heightened sensitivity to rejection (Cain et al. 2017), and from their therapist, which may pose difficulties in building therapeutic alliance (Gómez Penedo et al. 2019; Renner et al. 2012). Future research should investigate how treatments can be adapted to patients' interpersonal styles. For example, fostering patient‐therapist complementarity in communal behaviors (i.e., warmth) may strengthen alliance early in treatment (Gómez Penedo et al. 2021).

### 4.4 Strengths and Limitations

In the present study we took a transdiagnostic approach, using data from a large, naturalistic cohort study of individuals with current and past anxiety and depressive disorders, spanning 9 years of data collection. This allowed us to examine the relevance of interpersonal functioning in depression and anxiety across a broad range of clinical and subclinical individuals and to include an assessment of chronicity. While prior longitudinal research on interpersonal style and chronicity has been limited to single disorders and smaller samples (e.g., Cain et al. 2012; Thomas et al. 2014), the present study extends this work transdiagnostically in a large cohort. The inclusion of individuals with past diagnoses who may have residual symptoms offers insights into interpersonal functioning in recurrent anxiety and/or depression in the absence of current disorders. Additionally, the inclusion of a comparison group that was never diagnosed with any of the examined anxiety and depressive disorders is a strength of this study as archival norm data of patients or students that are not necessarily demographically similar to the studied sample are typically used for SSM analyses using IIP data (e.g., Barrett and Barber 2007). To examine interpersonal subtypes, we opted for a quadrant‐based categorical approach to group individuals based on interpersonal style. This approach is clinically intuitive as its boundaries lie along the axes of Agency and Communion and it offers clear advantages over cluster analysis, including comparability across data sets and it avoids the instability and data‐specific variability often seen in cluster‐derived subtypes (Gurtman 1996; König et al. 2016). Additionally, it allows for a straightforward illustration of different styles in clinical practice compared to a dimensional approach.

Despite these strengths, there are also several limitations. First, interpersonal problems were assessed only once (similarly to prior research), which limits our ability to evaluate the stability of interpersonal problems over time. Future research should examine the interplay between symptoms and interpersonal problems using repeated assessments (Hopwood et al. 2021; Wright et al. 2023). Second, the interpersonal assessment took place after the diagnostic data to determine chronicity were collected. The retrospective nature of our chronicity assessment introduces uncertainty regarding causality. For example, chronic anxiety and/or depression may have shaped a cold‐submissive interpersonal style over time. Therefore, we cannot rule out reverse or bidirectional effects. Third, there are limitations regarding the generalizability of our comparison group to populations without any lifetime diagnoses of mental disorders: (1) they expressed interest in participating in a longitudinal study on anxiety and depression, (2) they were compliant with six waves of data collection over a 9‐year period, and (3) a subset exhibited subthreshold anxiety and depression symptoms at baseline (Penninx et al. 2008). Additionally, (4) even though at baseline individuals with a primary diagnosis of obsessive‐compulsive disorder, psychotic disorder, bipolar disorder, or severe addiction were not eligible to participate in NESDA, we cannot exclude the emergence of these or other mental health conditions since the baseline assessment. Despite these limitations, we still found meaningful differences in interpersonal functioning between currently diagnosed individuals and our comparison group, whose characteristics likely reduced, rather than inflated, these observed differences. Fourth, the quadrant‐based approach to examine interpersonal subtypes has numerous advantages, but it also simplifies the interpersonal circumplex into categories. Dimensional models were shown to be statistically superior in modeling interpersonal problems than categorical approaches (Wendt et al. 2019). Indeed, the goodness‐of‐fit of the cold‐dominant quadrant's (Q2) observed profile to the sinusoidal curve was inadequate in the present study (< 0.70; Wright et al. 2009; Zimmermann and Wright 2017), which means comparisons to this quadrant in terms of angular displacement are not fully interpretable (Zimmermann and Wright 2017). Additionally, the relatively small cell sizes of Q2 likely contributed to wider credible intervals and greater uncertainty in estimates for comparisons with this quadrant. While the pattern of results remained consistent across sensitivity analyses including demographic covariates, findings involving Q2 should be interpreted with caution.

## 5 Conclusion

This study expands the existing body of knowledge by using data from a large longitudinal cohort study to examine how interpersonal problems relate to anxiety and depression over time in currently symptomatic and non‐symptomatic individuals. In line with previous research, we found that individuals with anxiety and/or depressive disorders experience more general interpersonal distress and tend to be more submissive than those who have never been diagnosed with anxiety and/or depression, and also compared to those with a past diagnosis. While this suggests that a submissive interpersonal style may contribute to or at least maintain anxiety and depression, interpersonal subtypes varied substantially within patient and comparison groups. We confirmed that a cold‐submissive interpersonal style was associated with greater comorbidity and chronicity. Our findings provide reasonable evidence for the concept of interpersonal pathoplasticity and highlight the potential clinical value of considering interpersonal style in prognosis and treatment planning. However, determining whether interpersonal style functions as a risk factor for comorbid and chronic disorder trajectories will require prospective studies in which interpersonal functioning is assessed prior to clinical outcomes.

## Author Contributions

**Jacqueline Peters:** conceptualization, formal analysis, methodology, visualization, writing – original draft preparation, writing – review and editing, project administration. **Peter J. de Jong:** conceptualization, methodology, resources, writing – review and editing. **Marije aan het Rot:** conceptualization, methodology, funding acquisition, writing – review and editing.

## Conflicts of Interest

The authors declare no conflicts of interest.

## Supporting information


Supporting File

## Data Availability

The *Netherlands Study of Depression and Anxiety* (NESDA) is an ongoing cohort study and we are not authorized to share any study data online. Access to NESDA data can be obtained by submitting a research proposal, please see (nesda.nl) for the data sharing policy and information on how to request data. Supporting materials and R scripts for data analysis, tables, and figures can be found on the Open Science Framework website of this study (osf.io/6e4gs).

## Footnotes

1 Although participants without a lifetime diagnosis of the examined disorders serve as a comparison group in this context, these individuals expressed interest in participating in a study on anxiety and depression, and a subset of them exhibited subthreshold symptoms at baseline (Penninx et al. 2008).

2 The exact sample size differed by analysis due to missing data in the assessment of anxious and depressive symptom severity.

3 Information on gender identity is not available in NESDA.

4 We could not calculate internal consistency for the QIDS and BAI, because we did not have access to individual item scores for these questionnaires.

5 This Gaussian model (default in Bayesian regressions with brms) does not perfectly fit the observed distribution, but it converged and provided stable estimates where the more theoretically appropriate ordinal models failed to converge. Converging issues are likely related to the low sample size in the “agentic” quadrants among currently diagnosed individuals (Q1: *n* = 40, Q2: *n* = 8).

6 Note that a recent meta‐analysis found only general interpersonal distress, but not Agency or Communion, to be associated with poorer treatment outcomes (Gómez Penedo et al. 2025), while the association of the interaction of Agency and Communion (akin to interpersonal style) with treatment outcome was not assessed. More research on this topic is thus needed.
