# Supplementary material for: Distant and Distressed: Interpersonal Problems in Anxiety and Depression
Source: J Clin Psychol. 2026 Sep 12;82(12):1977–89. doi: 10.1002/jclp.70212 (PMC13733650; doi:10.1002/jclp.70212)
Supplement: Supplementary file 1 [file JCLP-82-1977-s001.pdf]

Supplementary Materials

Distant and distressed:

Interpersonal problems in anxiety and depression

These supplementary materials, as well as R scripts for data analysis, tables, and figures can be found on the Open Science Framework website of this study (osf.io/6e4gs).

S1: The Interpersonal Circumplex and its Quadrant-Based Interpersonal Styles

Interpersonal Subgroups Based on the Quadrants of the Interpersonal Circumplex (Full sample)

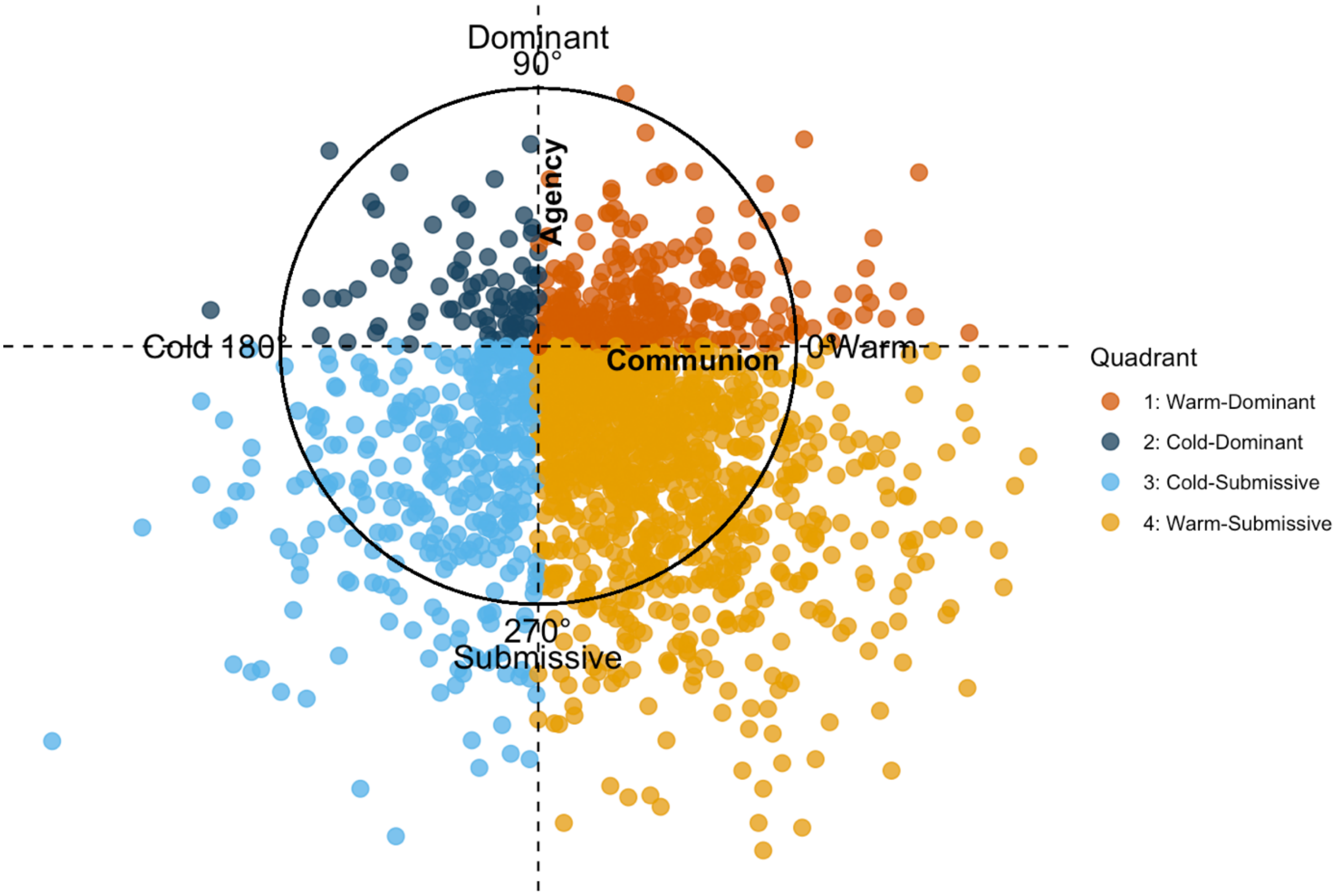

Note. N = 1800. Angular displacement based on the Structural Summary Method was used to calculate the degrees that the quadrants are based on. Warm-dominant quadrant (Q1): n = 299. Cold-dominant quadrant (Q2): n = 78. Cold-submissive quadrant (Q3): n = 348. Warm-submissive quadrant (Q4): n = 1075.

Interpersonal Subgroups Based on the Quadrants of the Interpersonal Circumplex (Subset With a Current Diagnosis)

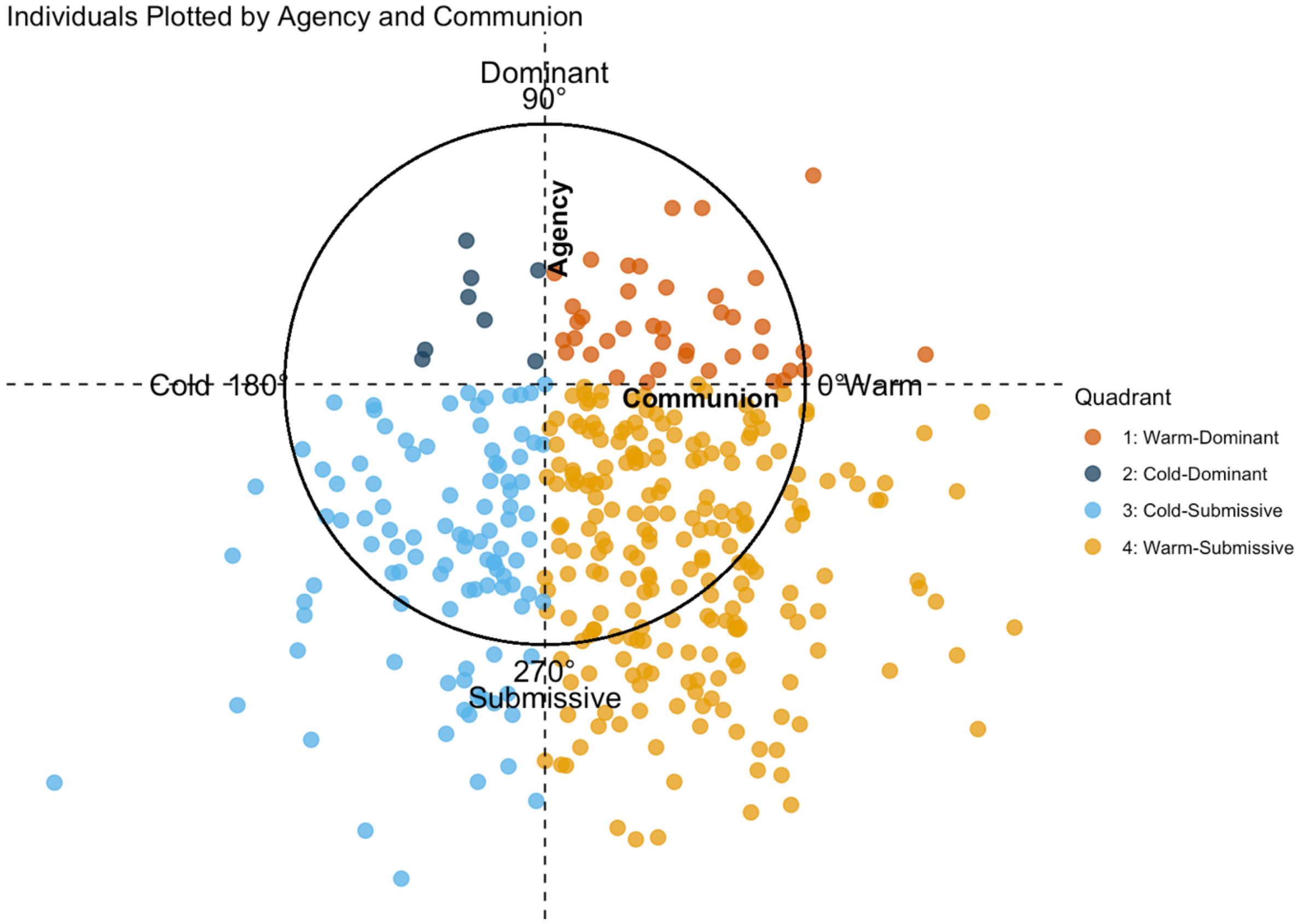

Note. n = 371. Currently diagnosed subset. Angular displacement based on the Structural Summary Method was used to calculate the degrees that the quadrants are based on. Warm-dominant quadrant (Q1): n = 40. Cold-dominant quadrant (Q2): n = 8. Cold-submissive quadrant (Q3): n = 102. Warm-submissive quadrant (Q4): n = 221.

S2: Supplemental Diagnostic Information

Diagnostic Information Among Individuals With a Current Diagnosis of a Depressive, and/or Anxiety Disorder

	Total		Depression-only		Anxiety-only		Comorbid Anxiety & Depression	
Disorder	n	%	n	%	n	%	n	%
Dysthymia	72	19.4	27	27.6	0	0.0	45	46.9
Major Depressive Disorder	170	45.8	83	84.7	0	0.0	87	90.6
Social Phobia	128	34.5	0	0.0	80	45.2	48	50.0
Panic Disorder with Agoraphobia	52	14.0	0	0.0	26	14.7	26	27.1
Panic Disorder without Agoraphobia	42	11.3	0	0.0	26	14.7	16	16.7
Agoraphobia without panic disorder	68	18.3	0	0.0	50	28.2	18	18.8
Generalized Anxiety Disorder	63	17.0	0	0.0	28	15.8	35	36.5
Total participants	371		98		177		96	

Note. Specific diagnoses among participants with a current diagnosis, then split by a current diagnosis of depression-only (i.e., dysthymia, major depressive disorder, or both, but no anxiety disorder), anxiety-only (i.e., at least one anxiety disorder, but no depressive disorder), or comorbid anxiety and depression (i.e., at least one anxiety and depressive disorder). Current diagnosis is defined as meeting the diagnostic criteria in the past month.

Total: All participants with a current diagnosis. Totals indicate the sum of individuals in each category (anxiety-only, depression-only, comorbid anxiety and depression), which may not be the sum of diagnoses in each column, as comorbid diagnoses are possible. Only diagnoses of panic disorder with agoraphobia, panic disorder without agoraphobia, and agoraphobia without panic disorder are mutually exclusive.

Percentages indicate how many individuals in each category (anxiety-only, depression-only, comorbid anxiety and depression) meet the diagnostic criteria for a specific diagnosis, e.g., 46.9 % of individuals with comorbid anxiety and depression have a current diagnosis of dysthymia and at least one current anxiety disorder ((45/96)x100 = 46.9%), whereas 90.6% ((87/96)x100 = 90.6%) of those with comorbid anxiety and depression have a diagnosis of major depressive disorder and at least one current anxiety disorder. As multiple diagnoses are possible, percentages can add up to more than 100% for each column, e.g., 27.6% of those with depression-only have dysthymia while 84.7% have major depressive disorder, which implies that 12.3% ((27.6%+84.7%)-100%) of individuals with depression-only have both dysthymia and major depressive disorder.

S3: Outlier Screening

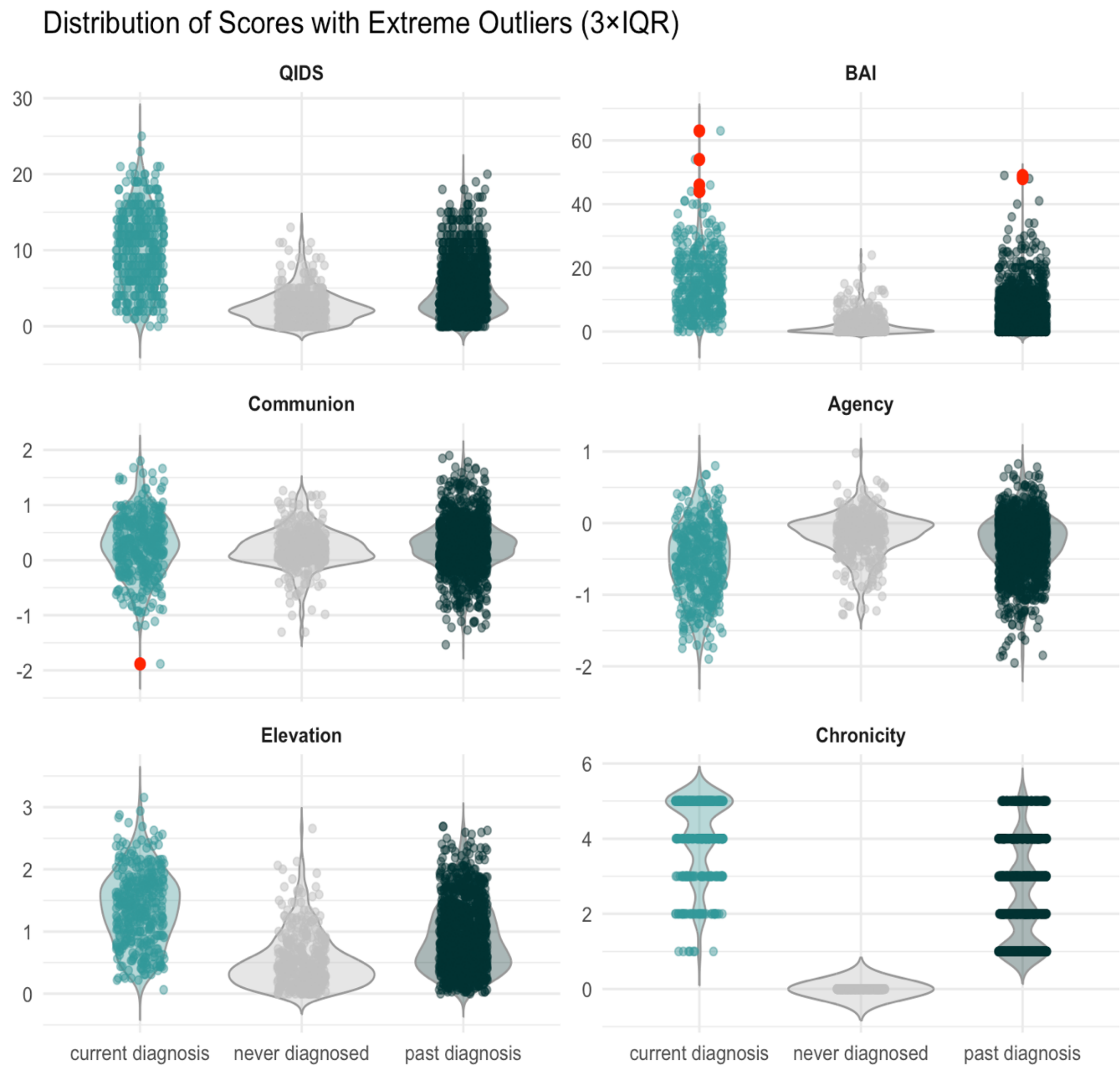

Note. Extreme outliers exceeding 3 times the interquartile range are marked in red.

S4: Descriptives Means and SDs for Currently Diagnosed Subset, Split by Quadrant-Based Interpersonal Style

**Interpersonal style**		Q1: Warm-dominant	Q2: Cold-dominant	Q3: Cold-submissive	Q4: Warm-submissive	BF_{10}
Age	M (SD)	53.52 (12.21)	51.12 (15.23)	52.87 (11.23)	50.1 (11.8)	0.75
	n	*n = 40*	*n = 8*	*n = 102*	*n = 221*	
QIDS	M (SD)	9.03 (4.86)	10.75 (3.15)	10.84 (5.05)	9.71 (5.06)	0.08
	n	*n = 40*	*n = 8*	*n = 102*	*n = 221*	
BAI	M (SD)	13.5 (10.75)	11.5 (8.3)	15.59 (9.98)	15.26 (9.88)	0.11
	n	*n = 40*	*n = 8*	*n = 101*	*n = 220*	
Chronicity	M (SD)	3.8 (1.2)	3.62 (0.92)	4.34 (0.98)	4.06 (1.11)	20.18
	n	*n = 40*	*n = 8*	*n = 102*	*n = 221*	
Agency	M (SD)	0.24 (0.19)	0.29 (0.17)	-0.66 (0.43)	-0.66 (0.43)	7.78×10^{30}
	n	*n = 40*	*n = 8*	*n = 102*	*n = 221*	
Communion	M (SD)	0.52 (0.33)	-0.26 (0.17)	-0.4 (0.33)	0.54 (0.38)	1.66×10^{65}
	n	*n = 40*	*n = 8*	*n = 102*	*n = 221*	

Note. Values are means with standard deviations in parentheses. n = number of participants per group. QIDS: Quick Inventory of Depressive Symptomatology (range: 0-27). BAI: Beck Anxiety Inventory (range: 0-63). Chronicity: Number of follow-ups where anxiety and/or depressive disorder diagnosis was present (range 0-5). Dimensional scores of Agency and Communion. BF_{10}: Bayes Factor. $BF_{10} > 1$ indicates that the data are more likely under the model including a main effect compared to under the null model, using Bayesian regressions.

S5: Correlations Between General Interpersonal Distress, Symptom Severity, Chronicity, Agency, and Communion

Full Sample

Variable	Age	Elevation	QIDS	BAI	Chronicity	Agency	Communion
Age	—						
Elevation	0.14***	—					
QIDS	0.11***	0.58***	—				
BAI	0.03	0.52***	0.67***	—			
Chronicity	-0.01	0.53***	0.56***	0.57***	—		
Agency	0.01	-0.49***	-0.32***	-0.31***	-0.33***	—	
Communion	-0.01	0	0.01	0.06**	0.02	-0.07**	—

Note. Spearman's correlations with Bonferroni correction. * p < .05, ** p < .01, *** p < .001. QIDS: Quick Inventory of Depressive Symptomatology (range: 0-27). Elevation: Structural Summary Method parameter, reflecting general interpersonal distress. BAI: Beck Anxiety Inventory (range: 0-63). Chronicity: Number of follow-ups where anxiety and/or depressive disorder diagnosis was present (range 0-5). Dimensional scores of Agency and Communion. N = 1800, except for associations with BAI (n = 1795).

Subsample With a Current Diagnosis of an Anxiety and/or Depressive Disorder

Variable	Age	Elevation	QIDS	BAI	Chronicity	Agency	Communion
Age	—						
Elevation	0.09	—					
QIDS	0.06	0.46***	—				
BAI	-0.01	0.3***	0.56***	—			
Chronicity	0.08	0.3***	0.33***	0.35***	—		
Agency	0.09	-0.41***	-0.2***	-0.15**	-0.17***	—	
Communion	-0.1*	-0.23***	-0.08	0.02	-0.07	0.02	—

Note. Spearman's correlations with Bonferroni correction. * p < .05, ** p < .01, *** p < .001. QIDS: Quick Inventory of Depressive Symptomatology (range: 0-27). Elevation: Structural Summary Method parameter, reflecting general interpersonal distress. BAI: Beck Anxiety Inventory (range: 0-63). Chronicity: Number of follow-ups where anxiety and/or depressive disorder diagnosis was present (range 0-5). Dimensional scores of Agency and Communion. N = 371, except for associations with BAI (n = 369).

S6: Correlation-Based Structural Summary Statistics for Anxiety and Depression Severity

Correlation-Based Structural Summary Statistics with 95% CIs for the Beck Anxiety Inventory (BAI) and the Quick Inventory of Depressive Symptomatology (QIDS)

	Elevation	Amplitude	Displacement	R^2
BAI	0.33 (0.30, 0.37)	0.09 (0.06, 0.11)	288.6 (271.5, 307.5)	0.794
QIDS	0.41 (0.38, 0.43)	0.11 (0.09, 0.14)	269.4 (257.3, 281.9)	0.868

Radar chart (A) and curve plot (B) of diagnostic status projected into the Interpersonal Circumplex Model

A B

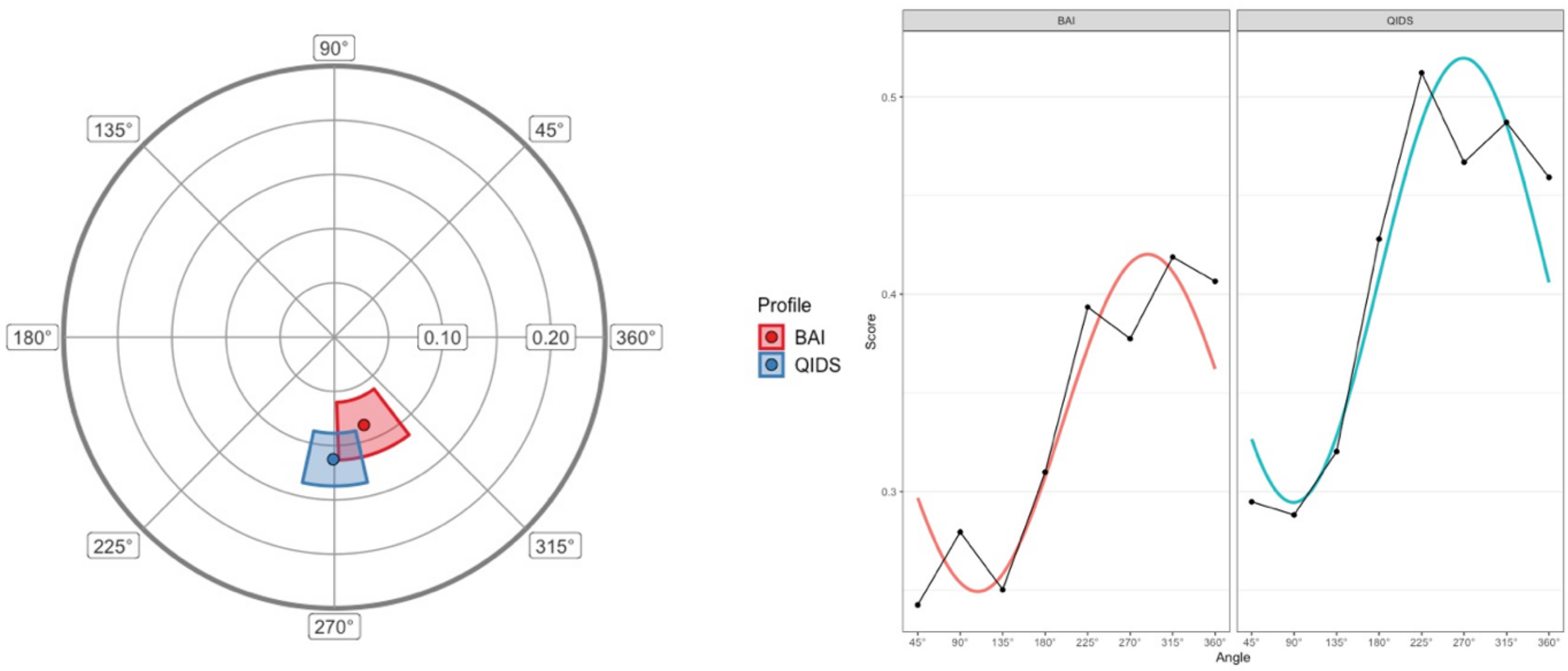

Note. Based on the Inventory of Interpersonal Problems. BAI: Beck Anxiety Inventory. QIDS: Quick Inventory of Depressive Symptomatology. **A:** Dots represent mean Amplitude and Displacement values and coloured regions reflect the corresponding 95% confidence intervals. **B:** Curve plots indicate the goodness of fit of the modelled to the observed data. N = 1800.

S7: Frequency Tables for Sex and Marital Status

Frequency Table of Sex and Marital Status, Split by Current Diagnostic Status

Variable		current diagnosis	never diagnosed	past diagnosis	Total
Sex	female	254 (68.5%)	211 (57.7%)	724 (68.1%)	1189 (66.1%)
	male	117 (31.5%)	155 (42.3%)	339 (31.9%)	611 (33.9%)
Marital Status	Divorced	53 (14.3%)	23 (6.3%)	149 (14%)	225 (12.5%)
	Living apart	10 (2.7%)	5 (1.4%)	17 (1.6%)	32 (1.8%)
	Married	177 (47.7%)	200 (54.6%)	516 (48.5%)	893 (49.6%)
	Never been married	125 (33.7%)	119 (32.5%)	341 (32.1%)	585 (32.5%)
	Widow(er)	6 (1.6%)	19 (5.2%)	40 (3.8%)	65 (3.6%)

Note. Diagnostic status is based on current, past, and no lifetime (i.e., never diagnosed) diagnoses with any of the examined anxiety and depressive disorders.

Frequency Table of Sex and Marital Status, Split by Quadrant-Based Interpersonal Style

Variable		1: Warm-dominant	2: Cold-dominant	3: Cold-submissive	4: Warm-submissive	Total (n/%)
Sex	female	160 (53.5%)	36 (46.2%)	178 (51.1%)	815 (75.8%)	1189 (66.1%)
	male	139 (46.5%)	42 (53.8%)	170 (48.9%)	260 (24.2%)	611 (33.9%)
Marital Status	Divorced	40 (13.4%)	17 (21.8%)	38 (10.9%)	130 (12.1%)	225 (12.5%)
	Living apart	6 (2%)	1 (1.3%)	7 (2%)	18 (1.7%)	32 (1.8%)
	Married	160 (53.5%)	33 (42.3%)	160 (46%)	540 (50.2%)	893 (49.6%)
	Never been married	89 (29.8%)	26 (33.3%)	125 (35.9%)	345 (32.1%)	585 (32.5%)
	Widow(er)	4 (1.3%)	1 (1.3%)	18 (5.2%)	42 (3.9%)	65 (3.6%)

S8: Structural Validity of the Inventory of Interpersonal Problems

Before conducting our confirmatory analyses, we examined the structural validity of the IIP. First, we tested whether the correlation between Communion and Agency approached zero, as these dimensions are assumed to be orthogonal in the interpersonal circumplex. Second, we calculated supplementary correlations between Communion and Agency and each interpersonal subscale to evaluate whether the interpersonal octants mapped onto their expected locations in the circular space (Soldz et al., 1995). Octant subscales of the IIP were calculated by taking the average of the four items in each subscale. If an item was missing, then the average of the remaining items was taken[1]. Lastly, we report the goodness-of-fit (R^2) from all SSM group analyses, which indicate how well the data conform to the expected circumplex structure.

We found that the correlation of Communion and Agency was -.07 and thus close to zero, as expected. Correlations between Communion and Agency with the octant subscales of the IIP as well as their visualizations can be found underneath. The overall order of the octant scales in the circular space was maintained, but BC (vindictive/ cold-dominant) did not correlate significantly with Agency. Additionally, theoretically warm (LM: overly nurturant) and cold (DE) subscales showed some association with low Agency, instead of no association. Nonetheless, the goodness of fit of the modelled curve using SSM to the observed data for the whole sample was good (R^2 = .89). Taken together, the IIP approached the theoretically proposed circular structure reasonably well.

[1] We would have excluded participants if >3 items were missing within one subscale, but there were none, other than the participant that was already excluded from our study for missing 11 items.

Associations Between Octant Scores of the Inventory of Interpersonal Problems with the Dimensions of Agency and Communion

IIP Octant	r (Communion)	r (Agency)
PA	0.07	0.15
BC	-0.46	-0.08
DE	-0.49	-0.30
FG	-0.35	-0.55
HI	0.07	-0.76
JK	0.27	-0.69
LM	0.46	-0.38
NO	0.31	0.12

Note. Spearman correlations of the octant subscales of the Inventory of Interpersonal Problems with Communion (X) and Agency (Y). PA = Domineering; BC = Vindictive; DE = Cold; FG = Socially Avoidant; HI=Non-assertive; JK=Exploitable; LM=Overly Nurturant; NO=Intrusive.

Subscale Projections for the IIP in the Circular Space

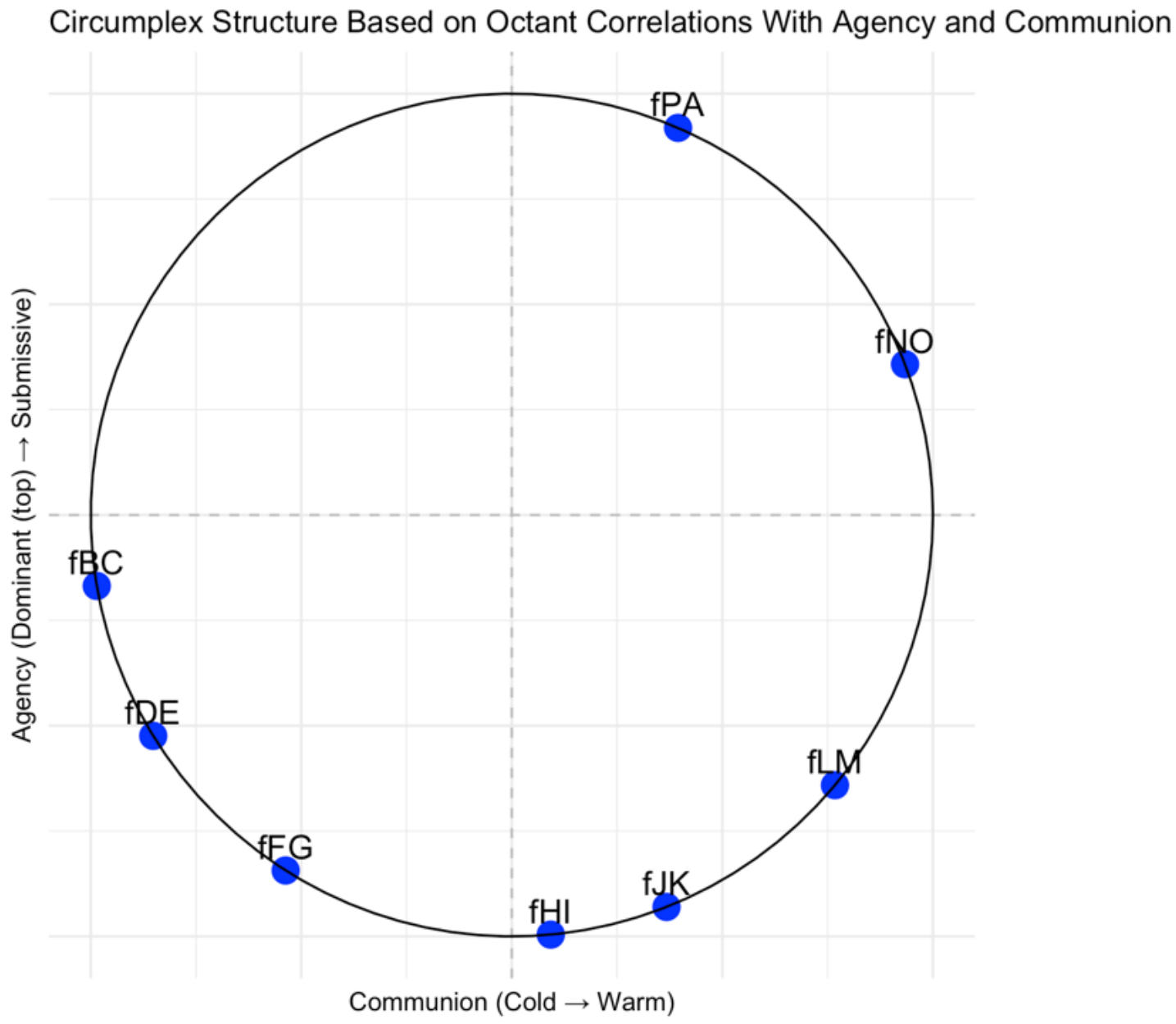

Note. IIP: Inventory of Interpersonal Problems. PA = Domineering; BC = Vindictive; DE = Cold; FG = Socially Avoidant; HI=Non-assertive; JK=Exploitable; LM=Overly Nurturant; NO=Intrusive.

S9: Radar Chart (A) and Curve Plot (B) of Quadrant-Based Interpersonal Styles Into the Interpersonal Circumplex Model

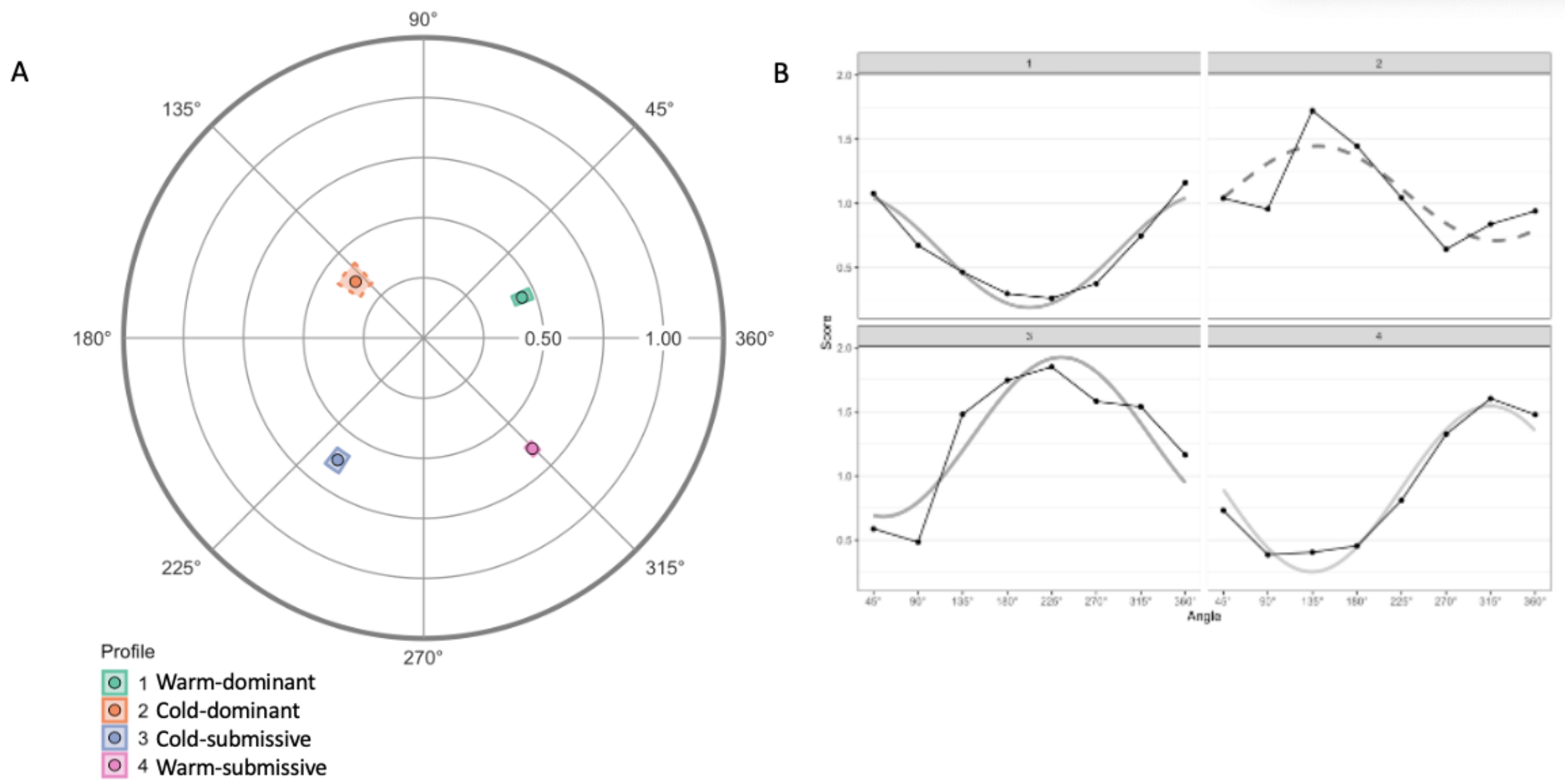

Note. Based on the Inventory of Interpersonal Problems. **A:** Dots represent mean Amplitude and Displacement values and coloured regions reflect the corresponding 95% confidence intervals. **B:** Curve plots indicate the goodness of fit of the modelled to the observed data.

Reference

Soldz, S., Budman, S., Demby, A., & Merry, J. (1995). A short form of the inventory of interpersonal problems circumples scales. *Assessment*, *2*(1). https://doi.org/10.1177/1073191195002001006
